# Safety and Efficacy of Ultrasound-Guided Perineural Hydrodissection as a Minimally Invasive Treatment in Carpal Tunnel Syndrome: A Systematic Review

**DOI:** 10.3390/jpm14020154

**Published:** 2024-01-30

**Authors:** Valerio Sveva, Giacomo Farì, Annatonia Fai, Alessio Savina, Mattia Giuseppe Viva, Francesco Agostini, Maurizio Ranieri, Marisa Megna, Massimiliano Mangone, Marco Paoloni, Andrea Bernetti

**Affiliations:** 1 Department of Anatomical and Histological Sciences, Legal Medicine and Orthopedics, Sapienza University, 00189 Rome, Italy; valerio.sveva@uniroma1.it (V.S.); alessio.savina@uniroma1.it (A.S.); mattiagiuseppe.viva@uniroma1.it (M.G.V.); francesco.agostini@uniroma1.it (F.A.); massimiliano.mangone@uniroma1.it (M.M.); marco.paoloni@uniroma1.it (M.P.); 2 Department of Biological and Environmental Sciences and Technologies (DiSTeBA), Università del Salento, 73100 Lecce, Italy; giacomo.fari@unisalento.it (G.F.); andrea.bernetti@unisalento.it (A.B.); 3 Department of Translational Biomedicine and Neuroscience (DiBraiN), Aldo Moro University, 70121 Bari, Italy; maurizio.ranieri@uniba.it (M.R.); marisa.megna@uniba.it (M.M.)

**Keywords:** ultrasound-guided hydrodissection, carpal tunnel syndrome, entrapment mononeuropathy, median nerve, ultrasound-guided injections, neuropathic pain

## Abstract

Ultrasound-guided perineural hydrodissection (HD) is a novel technique that has been found to be effective in providing mechanical release of perineural adhesions and decompression of the nerve, reducing inflammation and edema and restoring its physiological function. It has a significant impact on chronic neuropathic pain (20 ± 4 weeks with VAS < 5 or VAS diminished by 2 points after the procedure). Carpal tunnel syndrome (CTS) is a common entrapment mononeuropathy, and its distribution is typically innervated by the median nerve. Patients with mild or moderate CTS may benefit from nonsurgical treatments or conservative therapies. This review was conducted following the preferred reporting items for systematic reviews and meta-analysis (PRISMA) statement guidelines. Four investigators assessed each title, abstract, and full-text article for eligibility, with disagreements being resolved by consensus with two experienced investigators. The qualitative assessment of the studies was carried out using the modified Oxford quality scoring system, also known as the modified Jadad score. Furthermore, risk of possible biases was assessed using the Cochrane collaboration tool. The results of this review suggest that US-guided HD is an innovative, effective, well-tolerated, and safe technique (11 out of 923 patients had collateral or side effects after the procedure). However, further studies comparing all drugs and with a larger sample population are required to determine the most effective substance.

## 1. Introduction

Carpal tunnel syndrome (CTS) is the most common nerve entrapment in the hand and wrist with an estimated lifetime prevalence of approximately 10%, and it is predominantly seen in women aged 50–54 and 75–84 years [1,2]. CTS is also considered the most costly and disabling upper-extremity musculoskeletal disorder in Western countries. It causes substantial expenses incurred via medical insurance, lost wages, diminished productivity, and increasing disability in affected individuals [3].

Although the precise pathophysiology of CTS remains unclear, it is believed that increased pressure within the carpal tunnel contributes to the interruption of nerve microcirculation, ischemia, impaired nerve conduction, increased vascular permeability, and disruption of axoplasmic flow, resulting in decreased function of the affected nerve [3]. This is attributed to the fixed volume of the carpal tunnel, an anatomical structure composed of bone and fibrous tissue [4].

**Figure 1:**
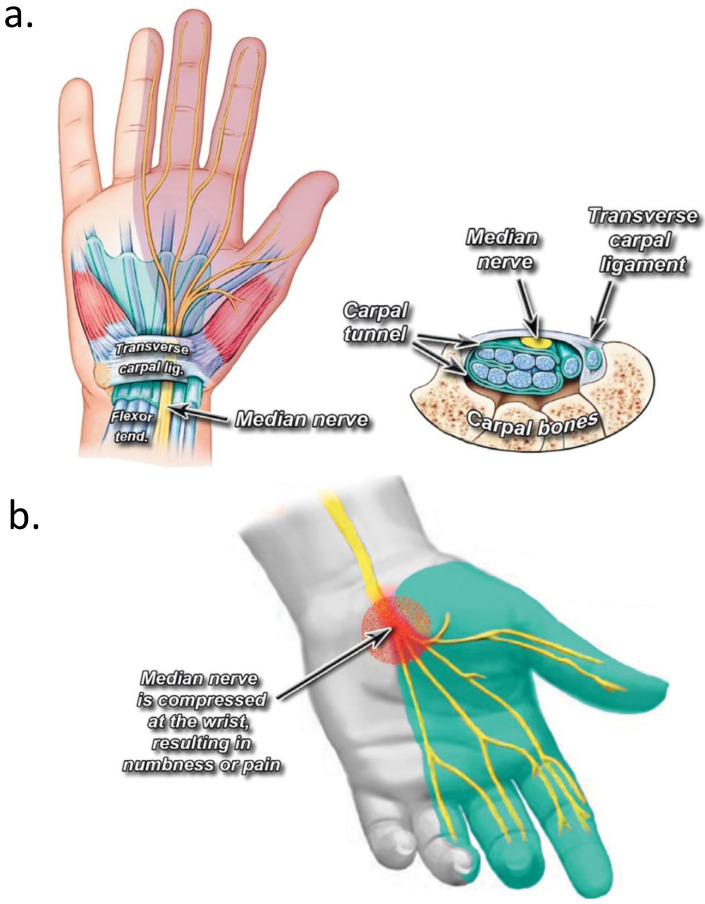
(**a**) Anatomy of the carpal tunnel; (**b**) median nerve’s site of compression in the carpal tunnel. Reprinted from Osiak et al., 2022 [7].

This increased pressure is often caused by mechanical, traumatic, inflammatory, or hormonal mechanisms, leading to unilateral or bilateral CTS, include diabetes, menopause, hypothyroidism, obesity, arthritis, acromegaly, pregnancy during the third trimester, distal radial fractures, and exposure to vibrating tools in the workplace [1]. Patients with these risk factors typically have pressure within the carpal tunnel ranging from 12 to 56 mmHg, compared to from 2 to 10 mmHg in healthy individuals [5]. Compression of the median nerve leads to the characteristic distribution of pain on the palmar aspect of the hand, which includes the volar aspect of the thumb, index finger, long finger, and radial volar half of the ring finger [6] (Figure 1).

The progression of CTS typically follows a specific sequence of symptoms, beginning with intermittent, nocturnal paresthesia and dysesthesia that increase in frequency and occur during waking hours. As the disease progresses, loss of sensation, weakness, and thenar muscle atrophy develop, resulting from extensive axonal degeneration [6].

CTS is primarily a clinical diagnosis, despite its well-known clinical presentation, and the optimal diagnostic approach remains uncertain [1]. Following a thorough patient anamnesis, a physical examination involving the first, second, and third sensory thumbs, along with positive findings of Tinel’s sign and/or Phalen’s maneuver, which reproduce the symptoms of CTS, can lead to a probable diagnosis of CTS. Additionally, evaluating the strength of muscles innervated by the median nerve is a typical examination during physical examination. A distinct sign of advanced CTS is atrophy of the abductor pollicis brevis (APB) or the inability to maintain the OK sign.

CTS may be misdiagnosed as C6–C7 radiculopathies or less commonly as lateral or medial cord plexopathy or proximal median nerve compression at the supracondylar process, caused by the presence of Struthers’ ligament or pronator syndrome [8,9].

The necessity of confirmatory testing in the context of CTS treatment decision-making is paramount. In this regard, electrophysiological assessment employing sensory and motor nerve conduction studies (NCSs) is highly sensitive in examining the median nerve dysfunction caused by demyelination and axonal loss. A neurophysiological classification of CTS severity has been established and utilized [10,11]. However, needle electromyography, which was previously considered an optional test, is more prone to excluding other concurrent diagnoses such as cervical radiculopathy [12].

An additional recent technical recommendation for confirming CTS is high-quality US of the wrist, capable of assessing the median nerve’s various components (fascicles, epineurium, and perineurium) and studying the surrounding structures. This imaging modality is employed when discrepancies exist between clinical examinations and electrophysiological tests [13]. US examination of the wrist is capable of identifying most secondary forms of CTS originating from high pressures within the carpal tunnel, revealing an increased cross-sectional area (CSA) of the nerve just proximally and distally at the site of compression [14].

The most effective treatment for CTS is multidisciplinary management, which can be broken down into several key strategies. Initially, modifications to daily habits, including the avoidance of heavy work-related activities and the adoption of appropriate biomechanical postures, should be considered as a first-line approach. This may involve the use of braces or splints at night, physiotherapy with exercises aimed at tendon and nerve gliding, low-power laser therapy, and local corticosteroid injections [15]. While conservative therapy can provide short-term relief for patients on pain and functional scales, its long-term effectiveness is still the subject of debate [16]. In cases of severe CTS, carpal tunnel release surgery may be necessary, which can be performed via conventional open or mini-open access or by ultrasound-guided decompression [17]. Despite treatment, some patients may experience persistent or recurrent symptoms of CTS, which can lead to the need for revision surgery due to factors such as incomplete decompression, perineural adhesions, excessive scarring, or the formation of a hematoma in the carpal tunnel [18].

The interest in US-guided perineural HD treatment for CTS has witnessed a substantial upsurge. This US-guided technique not only facilitates the mechanical release and decompression of the entrapped MN but also alleviates pain, improves symptoms, and promotes recovery in patients [19,20]. The decompression mechanism of US-guided HD is linked to the mechanical separation of the nerve from the surrounding soft tissues caused by the fluid flushing, resulting in a lower pressure on the nerve fascicles [21]. Furthermore, a diminishing pressure on the nervi nervorum, which constitute the intrinsic innervation of the nerve sheaths, produces a lesser stimulation of nociceptors and mechanoceptors, resulting in pain relief [22,23,24]. Additionally, another possible mechanism is that restoring the function of the vasa nervorum, which are small arteries that provide blood supply to the nerve bundles, would likely re-establish homeostasis, permitting the outflow of potential residual toxins, which are accumulated during the stasis and ischemia, and allowing nutrients and oxygen to enter inside the vessels [23,25]. All of these mechanisms act synergically on reducing the inflammation and edema of the nerve and surrounding tendons, restoring its physiological function. Various types of common injectates can be employed in HD, including normal saline (NS), corticosteroids, dextrose 5% (D5W), and platelet-rich plasma (PRP) [26]. Furthermore, an important innovation that has been analyzed is the use of ultrasound guidance, which allows for the surrounding nervous and tendon structures to not be damaged during the infiltration, as prior works have stated as a potential limitation of this procedure [27].

The objective of this systematic review was to assess the safety and efficacy of US-guided HD treatment for CTS using different agents, discuss the practical considerations regarding agent selection, and highlight the existing evidence in the literature.

## 2. Materials and Methods

### 2.1. Data Sources and Search Strategy

This systematic review was conducted following the preferred reporting items for systematic reviews and meta-analysis (PRISMA) statement guidelines [28]. The protocol was registered in PROSPERO (2023, n° CRD42023482770). The PubMed, Google Scholar, and Cochrane Library databases were utilized to identify scientific articles. Medical subject headings (MeSH) were employed as applicable. Candidate studies were identified through the use of the following Boolean search syntax: “(((“median nerve” OR “ultrasound-guided nerve” OR “hydrodissection”) AND (“neuropathic pain” OR “pain management”)) AND (clinical trial OR randomized controlled trial)).” The following filters were activated: text availability (full text), species (humans), languages (English), and period (last three years, 2020–2023). The search syntax employed for the PubMed database combined the MeSH database and Boolean search syntax. Additionally, the references of the collected articles were manually searched to identify any relevant publications.

### 2.2. Eligibility Criteria

The inclusion criteria were as follows: (1) Adults (>18 years old) with a neurophysiological or ultrasound and clinical diagnosis of mild-to-moderate or moderate-to-severe CTS. (2) Use of a perineural US-guided HD compared to a non-US-guided HD technique of the MN in patients with CTS, injecting a 0.9% NaCl solution or corticosteroid or a 5% dextrose solution or PRP. (3) Studies assessing changes in pain intensity (by visual analog scale (VAS) or numeric rating scale (NRS)), conducting patient-related outcome measures (PROMs) questionnaires, evaluating changes in clinical and functional symptoms (by the Boston carpal tunnel questionnaire (BCTQ)—symptom severity scale (SSS) and functional status scale (FSS)), or assessing changes in electrophysiological (sensitive and motor conduction velocities of MN, CMAP, and SNAP amplitudes) or US-guided features (CSA and echogenicity of the MN) as outcome measures after the US-guided HD injection in the carpal tunnel. (4) Retrospective or prospective randomized clinical trials (RCTs) in humans. (5) Articles written in English and published between 1 January 2020 and 1 November 2023.

The exclusion criteria were as follows: (1) studies with open surgery or endoscopic carpal release before HD of the MN in patients with CTS and (2) comments, expert opinions, case reports, case series, conference meeting abstracts, surveys, reviews, editorials, systematic reviews, meta-analyses, and letters.

### 2.3. Data Extraction and Outcome Measures

The studies retrieved using the search strategy were screened independently by 4 investigators (V.S., A.F., M.G.V., and A.S.) based on their titles and abstracts and by considering the inclusion and exclusion criteria. Then, the full text of eligible studies was further analyzed and independently assessed for eligibility. Any disagreement was resolved by consensus, asking 2 other experienced investigators (A.B. and F.A.). After inclusion, the study characteristics, research goals, and main findings were extracted and summarized. Moreover, the extracted information also included the study design, participant characteristics (number of participants, age, and gender), severity of CTS, follow-up interval after the US-guided HD procedure, and relative outcomes.

The main outcomes of interest were (1) pain, measured by the VAS or NRS scales; (2) clinical symptoms and functional features, measured by the Boston carpal tunnel questionnaire (BCTQ)—symptom severity scale (SSS) and functional status scale (FSS); (3) patient-related outcome measures (PROMs), assessed by subjective symptom changes and global assessment of treatment results; (4) electrodiagnostic findings, measuring sensory nerve conduction velocity (SNCV) and distal motor latency (DML); and (5) ultrasound measurement of nerve CSA.

### 2.4. Quality Assessment

Qualitative assessment of the studies was carried out using the modified Oxford quality scoring system, also known as the modified Jadad score [29]. It is a four-question scale assessing the randomization and concealment of treatment allocation groups, withdrawals and dropouts, the use of inclusion and exclusion criteria, and descriptions of statistical methods in the analyzed studies. Scoring was performed independently by the four aforementioned investigators. The modified Jadad score ranges from 0 to 5, and each question has a dichotomous answer (yes: 1 point; no: 0 points) [30,31]. A higher score indicates better RCT quality. If a study had a modified Jadad score >3 points, it was of a high quality; if the score was 2–3 points, it was of a moderate quality; and if the score was <2 points, it was of a low quality [29].

### 2.5. Risk of Bias Assessment

The risk of bias for all the included RCTs was assessed with the six domains defined by the Cochrane collaboration tool [32]. These six domains are: (1) selection bias due to random sequence generation and allocation concealment; (2) performance bias, with blinding of participants and personnel as a possible source of bias; (3) detection bias due to blinding of the outcome assessment; (4) attrition bias, evaluating possible incomplete outcome data; (5) reporting bias due to selective outcome reporting; and (6) other bias, evaluating any important concerns about bias not covered in the other domains. Each domain was judged as “low risk of bias” (“green”), “high risk of bias” (“red”), or “unclear risk of bias” (“yellow”). Finally, visualization of the authors’ judgments about each risk of bias item was presented both as percentages and as summary across all the included studies using the web app “robvis” [33].

## 3. Results

### 3.1. Identification of Studies

**Figure 2:**
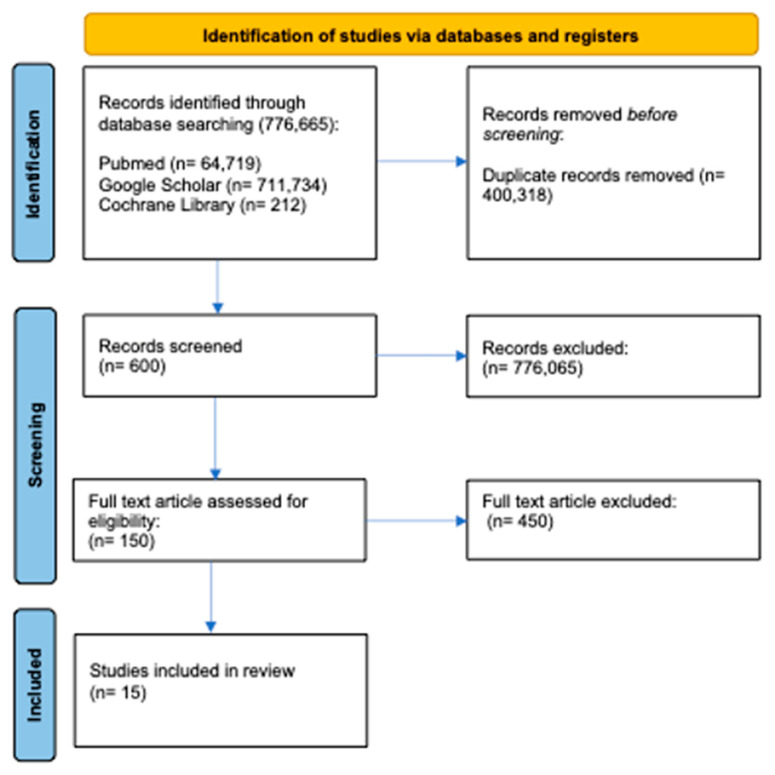
Study selection and eligibility screening flow according to PRISMA guidelines.

Studies were identified through a search of three databases (PubMed, Google Scholar, and the Cochrane Library). A total of 776,665 articles were extracted, with 64,719 coming from PubMed, 711,734 from Google Scholar, and 212 from the Cochrane Library. Studies were selected for inclusion based on their relevance to the review, and duplicates were excluded (*n* = 400,318). After removing review articles, unpublished studies, meta-analyses, case studies, practical guidelines, and books (*n* = 776,065), the full text of the remaining 150 articles was assessed for eligibility. Ultimately, 15 research articles were included in the review (Figure 2) [21,34,35,36,37,38,39,40,41,42,43,44,45,46,47].

### 3.2. Characteristics of Included Studies

The general characteristics of the studies included in this review are summarized in Table 1. Some studies focused on the efficacy of a single compound, while others compared multiple types of compounds for the treatment of CTS with HD. As a result, the findings are presented herein based on the type of compound administered. In total, 923 patients were included in this review. Specifically, five studies used corticosteroids as the primary treatment for HD [35,37,38,39,47]. Forogh et al. compared corticosteroid with oxygen–ozone (O2-O3) therapy in 40 patients with mild-to-moderate CTS [39]. Hsu and colleagues administered a corticosteroid injection after administering a local anesthetic (LA) in 126 patients [37]. Mezian et al. divided 46 patients into two groups and administered perineural or peritendinous injections of corticosteroids [38]. Finally, Wang et al. administered corticosteroid injections using perineural or intra-carpal injection in a group of 64 patients [47]. Santoso et al. administered a corticosteroid injection in one group of 15 patients and dextrose water (D5W) in another group of 15 patients, with a total of 30 patients injected [35]. D5W was also used as the principal type of injection in six studies to treat CTS [21,35,36,40,41,42]. As previously mentioned by Santoso et al., Li et al. evaluated 185 patients with CTS after D5W injection [41]. Lin et al. studied the efficacy of D5W using three different volumes in 39 patients divided into three groups [36]. He et al. conducted a study comparing the efficacy of combining D5W after corticosteroid injection in a control group of 24 patients who only received corticosteroids in a group of 25 patients [40]. Chao and colleagues treated 36 patients with persistent or recurrent CTS after primary surgery using D5W [42]. Wu and coworkers compared 16 patients treated with D5W to three groups of 15 patients each who were treated with NS, HA, and PRP, respectively [21]. Additionally, two studies evaluated the efficacy of PRP as an injection therapy for CTS [21,45]. The aforementioned study by Wu et al. has been cited; in contrast, Chen and colleagues treated 26 CTS patients with a single dose of PRP and compared the results with a control group that received only NS [45]. Continuing with another type of injected compound, two studies used NS as a solution for HD in CTS patients [34,43]. Chen and coworkers compared the efficacy of short-axis HD to long-axis HD injections for 57 patients with mild-to-moderate CTS using NS [43]. In a recent study, Huang et al. evaluated the most effective NS volume in 24 HD patients [34]. Finally, Su et al. compared the injection of HA with NS in 40 patients with mild-to-moderate CTS [44]. Kamel et al., on the other hand, compared NS injection with hyalase to NS injection alone in a cohort of 60 patients [42]. Moreover, nine articles were RCTs [34,36,38,39,43,44,45,46,47], five were retrospective studies [21,37,40,41,42], and one was a quasi-experimental study [35]. Undertaking a systematic review, we proceeded to evaluate all the outcome variables across all the studies that met our inclusion criteria. The BCTQ, comprising its two subscales FSS and SSS, was considered in 12 studies [21,34,35,36,37,38,39,40,43,44,46,47]. VAS or pain NRS was assessed in nine studies [21,35,36,38,39,40,42,44,46]. The diminishing US-CSA in wrists treated after HD was measured in eight studies [21,34,36,37,39,43,45,47]. Motor and sensitive nerve conduction studies (NCS), specifically DML and SNVC, were assessed in eight studies [21,34,36,39,43,44,45,47]. Only one study considered the QuickDASH scale [36], and another study used grip strength and 2-point discrimination as functional outcomes after HD procedures [38]. Lastly, two studies evaluated patient-reported outcomes (PROs) with subjective improvement of symptoms after US-guided injection [41,42].

Additionally, US-guided HD was performed in thirteen studies using an in-plane ulnar approach [21,34,35,37,38,39,40,41,42,43,44,45,46,47], using an in-plane radial approach in two studies [36,37], and using a long-axis approach in one study [44]. Complications or side effects, such as needling pain, subjective swelling or dizziness, electric shock sensation, or finger numbness that lasted <48 h, were assessed in all the selected studies, with only three studies mentioning some complications or side effects [36,37,47].

### 3.3. Evaluation of the Methodology and Credibility of the Studies

The methodological quality of the studies included in this systematic review was assessed using the modified Jadad score (as shown in Table 2). Two studies were classified as having a low risk of bias, with a score greater than 4 [38,44]. Additionally, eleven studies were found to have a moderate risk of bias, with a score ranging from 3 to 4 [21,34,36,37,39,40,41,42,43,44,45,46,47]. Only one study had a high risk of bias, with a score less than 3 [35].

### 3.4. Evaluation of Risk of Bias

**Figure 3:**
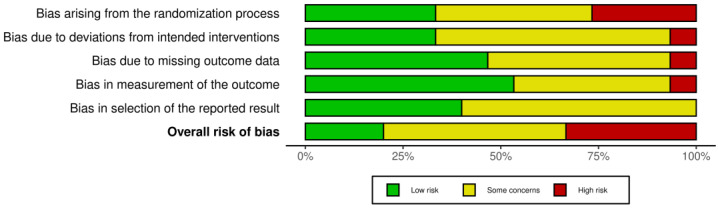
Risk of bias graph: review authors’ judgments about each risk of bias item presented as percentages across all included studies.

The risk of bias graph is reported in Figure 3. The overall level of risk of bias in all of the studies retrieved in this systematic review showed some concerns about the randomization process (selection bias), deviations from the intended intervention (performance bias), blinding of the outcome assessment (detection bias), incomplete measurement of the outcome data (attrition bias), and selective reporting of the results (reporting bias). More specifically, 47% of the studies highlighted some concerns, while 33% of them had a high risk of bias, and only 20% of them resulted having a low risk of bias.

**Figure 4:**
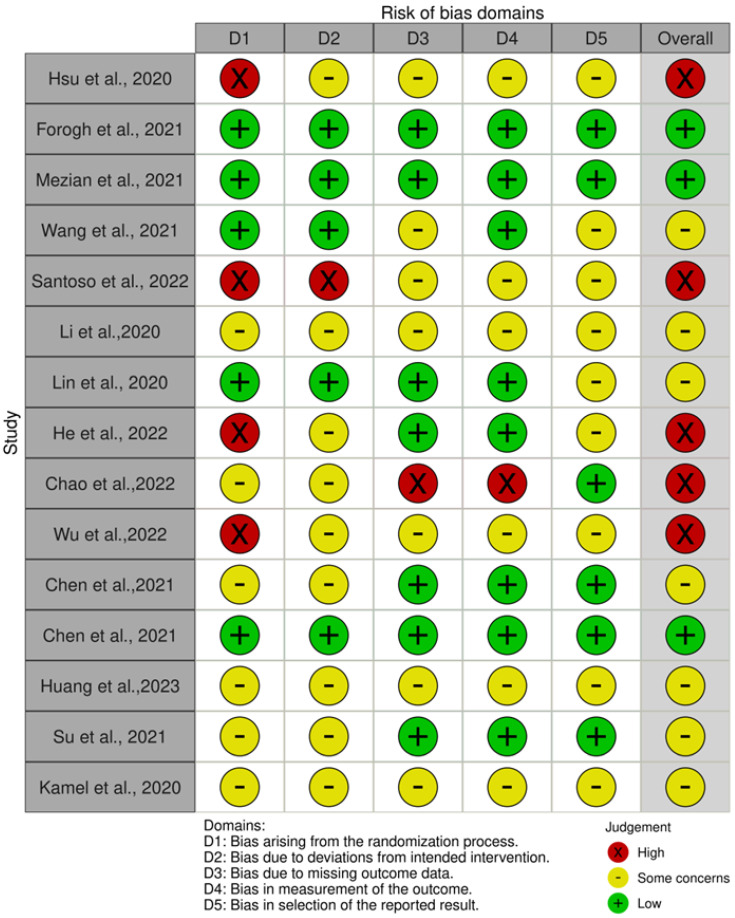
Risk of bias summary: review authors’ judgements about each risk of bias item for each included study [21,34,35,36,37,38,39,40,41,42,43,44,45,46,47].

Instead, a risk of bias summary is reported in Figure 4. It reveals that 5 out of the 15 studies presented a low risk of bias [36,38,39,43,47], and 4 of them presented a high risk of bias arising from the randomization process [21,35,37,40]. Conversely, 5 of the 15 studies showed a low risk of bias due to deviations from the intended intervention [36,38,39,43,47], and there was only 1 with a high risk [35]. Moreover, seven studies had a low risk of bias due to missing outcome data [36,38,39,40,43,44,45], and one study had a high risk [42]. Eight studies revealed a low risk of bias in measurement of the outcome [36,38,39,40,43,44,45,47], and only one study had a high risk [42]. Lastly, six studies presented a low risk of bias in selection of the reported results [38,39,42,43,44,45], and none had a high risk in this domain.

## 4. Discussion

This systematic review aimed to evaluate the effects of various injection compounds for US-guided HD in patients with CTS, the most prevalent entrapment neuropathy. To the best of our knowledge, previous systematic reviews have addressed this topic [3,20], but ours is the only one to consider and highlight the most recent studies conducted within the past three years. Although the pathophysiology of CTS has not been entirely clarified, increased pressure within the carpal tunnel is believed to be a probable cause of the disease, which may result from fibrosis of the connective tissue and adhesion of the flexor tendon synovial connective tissue around the MN [48]. At present, two forms of treatment strategies, conservative and surgical, are employed to alleviate CTS symptoms [49]. Conservative therapies, such as hand splinting, laser therapy, oral pharmacotherapy (e.g., acetyl-l-carnitine and/or gabapentinoids), physical therapy, therapeutic ultrasound, and musculoskeletal manipulations, are typically used for patients with mild-to-moderate CTS symptoms [6]. While these interventions provide short-term benefits, the effectiveness of long-term interventions remains a topic of debate. An additional interventional treatment that can be used alongside conservative therapies is local corticosteroid injection in the carpal tunnel at the wrist using US. This procedure aims to alleviate pressure within the tunnel by reducing inflammation and edema of the tendons passing through it [50]. However, the use of corticosteroid injection as a minimally invasive strategy for CTS is a matter of controversy. Although some studies have reported clinical improvements in patients with severe CTS at one month following corticosteroid injection, the effectiveness of this treatment in patients with mild-to-moderate CTS remains uncertain [51]. While one study indicated significant symptom relief within one month of corticosteroid injection, another study failed to demonstrate any symptom relief following the same treatment modality [52]. Surgical interventions for carpal tunnel syndrome (CTS) typically involve carpal tunnel release via the open approach, which can be the conventional open or mini-open release, or the endoscopic approach. This type of treatment is recommended for patients with severe CTS who have not experienced improvement with conservative treatment and is associated with acceptable long-term clinical outcomes [49]. However, complications can still lead to incomplete restoration of nerve function and poor patient-related and functional outcomes at long-term follow-up [53]. To address these issues, a new minimally invasive technique has been developed in the past decade. This technique involves using high-resolution ultrasound (US) to guide a needle inside the carpal tunnel without damaging the nerve or vascular structures, allowing for high-dose (HD) medication of the median nerve (MN). HD medication releases adhesions, liberates the nerve from surrounding tissues, and restores its physiological function. This is achieved through various mechanisms, including mechanical decompression and the pharmacological effects of the injectates, which improve functional outcomes, reduce pain, and aim to return the nerve conduction study (NCS) and ultrasound-controlled sphincterotomy (US-CSA) of the MN to physiological values.

Among the studies that were selected, all patients who were clinically suspected of having CTS received either an electrophysiological or ultrasound-based diagnosis of mild-to-moderate, moderate-to-severe, or severe CTS before undergoing US-guided HD treatment. As stated by Padua et al., the electrophysiological assessment had a cut-off value for SNCV of <3.6 msec and for DML of <4.3 msec, with a distance of 14 cm and 8 cm between the active recording and stimulation, respectively [10]. The electrophysiological study was used to classify the grade of CTS as normal, very mild, mild, moderate, severe, or very severe based on the results of the SNCV and DML tests [54]. The sensitivity and specificity of NCS for diagnosing CTS were reported to be 73.4% and 93.6%, respectively [55]. On the other hand, nerve ultrasound was used to assess the CSA of the MN at the proximal inlet of the carpal tunnel using electronic calipers [56]. The cut-off value for diagnosing CTS using nerve ultrasound was found to be 10–12 mm^2^, with a sensitivity and specificity of 77.6% and 86.8%, respectively [57]. The grading of CTS by ultrasonography was found to be strongly associated with electrophysiological measurements in mild, moderate, and severe cases, as reported in past studies [58,59]. Although NCS is the most commonly used method for diagnosing CTS, nerve US is increasingly being employed by physicians due to its ease of use and direct visualization of the nerve in the carpal tunnel. All of the selected studies used US-guided HD to compare different interventions with the aim of minimizing the collateral effect, increasing accuracy, and enhancing safety during the procedure, as any clinical effect differences would likely result from needle misplacement. In order to accurately assess the effectiveness of various injection techniques, it is essential to utilize US guidance. According to a study conducted by Evers and colleagues, the outcomes of US-guided injections were compared to those of blind injections in the treatment of CTS [60]. The results of this population-based cohort study showed that the difference in retreatment-free survival between the two groups was statistically significant, with the odds of injection failure within one year being 55% lower in the ultrasound-guided group than in the blind injection group.

The injected compounds used in the selected studies included corticosteroids, dextrose (D5W), platelet-rich plasma (PRP), normal saline (NS), hyaluronic acid (HA), and hyaluronidase. The mechanisms of these injectate compounds are described below.

Corticosteroids are anti-inflammatory agents that provide pain relief by inhibiting cytokines and reducing inflammatory mediators such as leukotrienes, prostaglandins, and platelet-activating factors. They also prevent the recruitment and activation of inflammatory cells such as lymphocytes, eosinophils, basophils, and macrophages and reduce edema by decreasing capillary permeability and blood flow [61]. Synthetic corticosteroid preparations for local injection have varying degrees of anti-inflammatory activity, solubility, and duration of action. Commonly used injectable steroids such as triamcinolone, methylprednisolone, and dexamethasone are derivatives of prednisolone. These compounds have an OH (hydroxyl) group, are glucocorticoid derivatives, and are readily available for use [62]. Corticosteroids have been shown to have a clinical effect in reducing pain and improving functional outcomes as well as decreasing CSA and providing more space around the nerve. A total of five studies utilized triamcinolone acetonide 40 mg/mL as the preferred corticosteroid for perineural injection [35,37,39,47]. One study used betamethasone [35], and one study used methylprednisolone 40 mg/mL [38]. Additionally, one study compared the efficacy of injecting 1 mL of triamcinolone acetonide versus 5 mL of D5W in terms of pain and functional outcomes, as mentioned previously [35].

D5W is an isotonic solution of dextrose in the form of D-glucose containing 278 mmol/L dextrose [63]. Its mechanism for relieving neuropathic pain after perineural injection remains debated. It has been proposed that D5W relieves pain through a sensorineural mechanism by downregulating transient receptor potential vanilloid receptor 1 (TRPV-1), which is often upregulated in cases of chronic neuropathic pain [64]. Another possible mechanism is a decrease in C-fiber activation by reversing the hypoglycemic status, which induces excessive C-fiber activation in the damaged nerve [65]. Despite its unclear mechanism, dextrose reduces pain and improves symptoms, function, electrophysiological findings, and CSA reduction [66]. Six studies used D5W with different volumes: two studies used 5 mL of D5W for US-guided HD [35,40]; two studies used 10 mL of the solution [41,42]; one study compared three different volumes for injection, 1 mL, 2 mL, and 4 mL [36]; and one study used 6 mL of D5W [21].

PRP is a portion of the plasma fraction of autologous blood with a high platelet concentration and contains numerous bioactive factors, including transforming growth factor-β, platelet-derived growth factor, and vascular endothelial growth factor, which can promote tissue repair and regeneration [67]. Once activated, secretory granules release many mediators important for homeostasis, growth factors, and cytokines affecting inflammation and angiogenesis, facilitating the natural healing process and promoting regeneration in many tissue types [68]. Because of PRP’s regenerative mechanism, this compound provides broad clinical effects, ranging from pain reduction to improving symptoms, function, electrophysiologic findings, and CSA reduction [20]. Only two studies used PRP with different volumes: one study used 3 mL and 5 mL [45], and the other used 6 mL of PRP [21].

Sodium chloride (NaCl), also known as 0.9% normal saline (NS), is a crystalloid fluid with an osmolarity of 30.8 mOsmol/L and a pH range of 4.5–7. It contains an equal amount of 154 mEq of sodium and chloride ions per 100 mL of injection [69]. It can be used as a standalone injectate or as a diluent for other injectates in hemodialysis (HD) procedures. Its primary purpose is to act as a perineural space expander without intrinsic inflammatory reduction or nerve repair effects [70].

Several studies have utilized NS for ultrasound (US)-guided HD procedures. One study evaluated the efficacy of 5 mL NS injected with different US positions of the probe (short-axis vs. long-axis) [43]. Another study used a volume of 6 mL of NS for HD [21]. A study compared the efficacy of injections of NS ranging from 5 mL to 10 mL [34]. Lastly, 2.5 mL of NS was compared to the same amount of hyaluronic acid (HA) injection in terms of efficacy of the HD treatment [44].

Overall, NS is a commonly used fluid in HD procedures and has been demonstrated to be effective in reducing resistance to longitudinal sliding of the median nerve, which can explain the improvement in pain and functional status [71].

Hyaluronic acid (HA) is a high-molecular-weight polysaccharide commonly found in the extracellular matrix of connective tissue. It can be derived from either animal or non-animal sources, with animal-derived HA sourced from rooster combs and non-animal-derived HA produced through the bio-fermentation of Streptococcus. The size of the molecule determines the lifespan of HA in particulate form, while the cross-linking density determines the longevity of HA, which allows for the production of a more concentrated and chemically and physically resistant form of HA. HA is anchored to the extracellular surface and acts as a scaffold to support cellular viability and health by maintaining molecules and proteins, influencing neurotransmission and signaling [72,73]. Previous animal studies have suggested that HA accumulates in demyelinating lesions, and while one study used 2.5 mL of HA for ultrasound-guided hemodialysis, compared to the same volume of normal saline, few studies have investigated the use of HA in this context [44].

Hyaluronidases are enzymes, classified as endoglycosidases, that possess the capability to degrade hyaluronic acid (HA) by depolymerization. In a study conducted by Kamel et al., the authors evaluated the efficacy of hyaluronidase combined with 10 mL of NS compared to the same volume of NS without hyaluronidase [46].

To critically assess the most effective injected compounds for treating CTS, the functional, ultrasonographic, and electrophysiological outcomes from the selected studies in this review were analyzed.

The BCTQ, comprising its FSS and SSS sub-components, serves as a reliable indicator of functional outcome improvement. VAS or NRS are commonly used to subjectively assess symptom amelioration or exacerbation post injection. In contrast, US or NCS are employed to evaluate the reduction in CSA at the carpal tunnel inlet as well as improvements in SNVC and DLM following hyaluronidase treatment.

Most studies demonstrated a decrease in BCTQ scores following corticosteroid injection, with some reporting statistically significant improvements at 2 weeks [37,38] that were maintained through 12 weeks of follow-up [38]. In another study, both the SSS and FSS subscales of the BCTQ exhibited significant decreases at 6- and 12-weeks post intervention, although hyaluronidase did not provide any additional benefits beyond perineural corticosteroid injection alone [47]. Indeed, based on the results of most studies, corticosteroid injections for CTS provide only short-term benefits [72,73,74]. Regarding the comparison of corticosteroid injection versus ozone injection (O2-O3), a study showed improvement in two subscales of the BCTQ (BCTQ-SSS/BCTQ-FSS) at weeks 6 and 12 after the injections, demonstrating the non-superiority of ozone compared to treatment with corticosteroids [39]. Regarding the comparison between corticosteroids and D5W, a study showed significant improvements in the FSS and SSS parameters before injection and 4 weeks after HD injection in both cases. The difference in FSS and SSS between patients injected with D5W and corticosteroids was not significant, indicating that the two compounds were equally effective [35]. Another study investigated the efficacy of HD with D5W as an add-on therapy after corticosteroid injection. Compared with the baseline data, both groups showed a greater improvement in BCTQ-SSS and BCTQ-FSS scores at different follow-up points. In addition, the subscale scores at week 4 were comparable between the steroid and combination groups (HD plus D5W). According to the authors, there were some limitations to be considered: the relatively small sample size, the short follow-up duration of injection, and, finally, the injection was performed twice in the combination group but only once in the corticosteroid group. A placebo effect may have existed in the combination group [40]. The utilization of different volumes of D5W and NS has demonstrated varying degrees of efficacy in the improvement of BTQS. A study comparing 1, 2, or 4 mL of D5W found that all groups showed significant improvements in BCTQ at all follow-up time points, with the 4 mL group exhibiting the greatest improvement at the 1st week, 4th week, and 12th week post injection. However, there was no significant difference between the groups at the 24th week post injection [36].

In contrast, a study comparing 5 mL and 10 mL volumes of NS found that the latter group outperformed the former in terms of improvement in SSS and FSS scores up to 24 weeks post injection. This suggests that a larger volume of NS may be more effective in improving symptoms [44].

Comparing NS and HA, a study found that HA was superior in terms of BTCQ at only the second week post injection. This suggests that HA may have a more immediate impact on improving BTQS compared to NS [46].

Eventually, a study was conducted to compare HD with an injection of catalase plus 10 mL of NS and a solution of HD with 10 mL of NS only. The results indicated that the group receiving catalase demonstrated significant improvements in symptom severity and functional status up to six months post injection, with continued improvement in SSS and FSS scores up to three months post injection. This suggests that the addition of catalase to the injection enhances its effectiveness in improving BTQS. In another study, patients in the saline group showed significant improvements in SSS and FSS at each follow-up. Additionally, significant improvements were observed in SSS scores at all time points in the PRP group compared to the control group. Lastly, a comparison was made between four injectates (NS, D5W, PRP, and HA). At the first and sixth months after injection, patients in all groups reported significant improvements compared to their baseline [45]. Overall, patients in the D5W, PRP, and HA groups showed more significant improvement than those in the NS group at all measured time points. For SSS, patients in the PRP group showed the greatest improvement, followed by those in the D5W group, while those in the HA group showed the least improvement. The difference in SSS improvement was significant in the first- (PRP > HA) and sixth-month (D5W > HA) values. Regarding FSS, the progressive trends between the three groups were similar to those of SSS, except for the sixth-month value between PRP and D5W, in which D5W was slightly better than PRP. Furthermore, the intergroup difference in FSS improvement reached significance (D5W > HA at the first and sixth months, respectively; PRP > HA at the first month) [21].

Pain relief was identified as the primary outcome measure in numerous studies included in this systematic review. The effectiveness of pain relief was assessed utilizing the VAS or NRS, yielding generally satisfactory outcomes. Injectable corticosteroids have demonstrated a significant reduction in pain, with an average decrease in VAS score of −2.5 ± 2.8 two weeks post injection, and the effect was sustained at 12 weeks [38]. In a comparison between ozone therapy and corticosteroids, Forogh et al. found that a single 3 mL injection of ozone at a concentration of 10 μg/mL was as effective in providing pain relief as corticosteroids [39]. Subjects who received D5W at various volumes demonstrated improved VAS scores [35,36,40,42]. Lin et al. discovered that 4 mL of D5W provided better efficacy at 1, 4, and 12 weeks, but after 24 weeks, the results for the three different volumes (1, 2, and 4 mL) were identical [36]. He et al. compared the use of corticosteroids and D5W in combination and found that this treatment resulted in a significant reduction in VAS scores at the 8- and 12-week follow-up, indicating that combination therapy is more effective than corticosteroid monotherapy in VAS [40]. Corticosteroid HD has been shown to be effective in providing pain relief, as assessed using the NRS 4 weeks after injection. Moreover, the same compound was more effective than D5W HD injections, which were equally effective [35]. Compared to saline, HA showed significantly superior outcomes in NRS, but only at the second week post injection, with uncertain clinical significance [44].

Regarding the results of ultrasonographic examinations, three studies reported a significant reduction in CSA after corticosteroid US-guided HD [36,38,39]. On the other hand, in the study by Wu et al., the decrease in CSA difference was more pronounced in the PRP group at 6 months compared to the D5W and HA groups [21]. This could be attributed to the anti-inflammatory and anti-edema effects of corticosteroids on the nerve fascicles as well as the delayed action of PRP in the nerve.

Regarding the outcomes of electrophysiological examinations, seven studies showed a statistically significant improvement in SNVC [34,38,39,43,44,45,47], and five studies showed an improvement in DML [36,38,39,45,47], with follow-up times ranging from 1 month to 12 months after US-guided HD. In all of the selected studies, SNVC and DML improved more when corticosteroids were used compared to HA, D5W, PRP, and NS. This result may be attributed to the anti-inflammatory and anti-edema effects of local corticosteroid injections on the neurophysiological function of the MN.

Another crucial topic for consideration is the approach for injection, which must be both safe and effective. The in-plane ulnar approach is preferred over the in-plane radial approach for US-guided HD. This approach offers several advantages. Firstly, the proximal part of the flexor carpi radialis tendon near the carpal tunnel is a safe area for injection, allowing physicians to better visualize the carpal tunnel structures around the nerve, which facilitates accurate perineural injection and prevents damage to the surrounding vessels, nerves, and tendons. Additionally, the needle tip and shaft can be visualized in-plane using this approach, allowing the physician to detach the surrounding connective tissues. In cases where difficulties are expected with the ulnar approach, such as persistent median artery or bifid median nerve, the radial approach can be used as an alternative. The radial approach can also avoid damaging the palmar cutaneous branch of the median nerve by clearly identifying the fascial passage of this nerve on US, as reported by Tagliafico et al. [75].

With regard to the collateral and adverse effects of the procedure, a rare complication is the occurrence of intraneural injection and associated neural swelling during HD. This can result in an increase in the cross-sectional area (CSA) of the median nerve (MN) at the carpal tunnel inlet. Therefore, measuring the CSA of the MN and assessing the linked clinical symptoms of the patient during US-guided HD are crucial in preventing this type of complication. In a previous study, Hsu et al. observed nine patients who experienced a long-lasting medial nerve injury with pain and sensory changes for more than 48 h after HD but recovered well at the 2-week follow-up. No patients in the study population reported permanent symptoms of median nerve injury or other complications, such as infections or allergic reactions, after HD injection. Three patients in the study reported vascular injury after the HD procedure in the carpal tunnel [37]. In another investigation, Wang et al. revealed that two individuals experienced mild discomfort following the injection, which dissipated without intervention within the first day [47]. Lastly, Wu et al. indicated that their patients experienced negligible discomfort during and immediately after the procedure, with no reports of adverse events in any patient [21]. All the other studies in this review emphasized that no patients reported adverse effects or complications during the procedure or during follow-up, as US-guided HD is a safe procedure, especially when the physician possesses ample experience with injections and musculoskeletal ultrasound.

A point that has to be elucidated is how to manage and carry-on further studies in this field of study. The call for further studies with larger sample populations is necessary for the scientific community and also for the physicians that, in their day-by-day working experience, require strong evidence-based knowledge that takes into account the interest of the patient. Moreover, homogeneity of the studied population is strictly recommended in order to avoid possible confounding results. Sample size calculation is highly recommended in order to detect a clinically important difference between groups. Eligibility criteria have to be very well thought-out, as do the randomization and allocation of interventional and control groups. For instance, random sequence generation and concealment allocation of participants within different groups could be managed using sealed envelopes given by a blinded investigator or using computerized random generator software used in commerce (i.e., https://www.randomizer.at/ (accessed on 30 December 2023); https://www.randomizer.org/ (accessed on 30 December 2023)). Then, performance bias could be reduced by using blinding investigators and standardized equipment for all the groups (i.e., a covered 5 mL syringe barrel with a 23-gauge needle), masking the different solutions used to inject into the carpal tunnel. Finally, it is important to report all the outcomes measured, highlighting statistically significant results with associated effect size calculation for a clear understanding of the results. Potential confounding factors could be avoided in terms of assessing pain and the use of NSAID’s before the procedure, administering and explaining well the questionnaires to the patients, reporting images captured by the sonographer for a clear visualization of procedure success, and repeating nerve conduction studies of the MN in the same laboratory in order to prevent different results that could undermine the study. Using this framework, future studies could achieve a good reproducibility and generalizability of the results, minimizing all the different risks of biases encountered previously.

Nonetheless, US-guided HD is a procedure that could be used by physicians in other different clinical settings (i.e., other mononeuropathies of the upper and lower limb like ulnar cubital syndrome [76], tarsal tunnel syndrome [77], or peroneal nerve entrapment at the fibular head [78]) and also in different patient populations (i.e., Morton’s neuroma [79] or the treatment of surgical scar tenderness [80]). A good result of the procedure is correlated with the experience and dexterity of the sonographer in terms of visualizing the point to inject into and the ability to introduce the needle without generating collateral effects or pain in the patient. For this reason, implementing a standardized sonographic checklist to assure that the point to inject is necessary [81,82] and using a cold spray for desensitizing the injection site could be a possible way to manage these practical challenges [83].

This systematic review was not without its limitations. Firstly, the selected studies varied greatly in terms of the interventions and comparisons as well as in terms of the follow-up periods and outcomes, resulting in substantial heterogeneity. Additionally, the varying injected volumes across studies could potentially impact clinical outcomes, as different modalities were used to decompress the wrist. Another factor to consider is the significant variability in CTS severity among the selected studies, along with the differences in participant demographics such as sex and age and the length of time since the onset of symptoms prior to treatment with US-guided HD. It is also worth noting that none of the studies assessed all the compounds described in this review, and there was no solid recommendation on which injectate is the best option for patients with CTS. As such, the decision is ultimately based on the physician’s discretion, taking into account that corticosteroids, NS, and D5W are more cost-effective than HA or PRP and may be considered as the first line of treatment for CTS with US-guided HD.

## 5. Conclusions

Patients are typically considered for carpal tunnel injection following the failure of conservative therapies, including wrist immobilization, physical therapy, and oral analgesic medications. US-guided HD techniques with various compounds may be used preoperatively to confirm the diagnosis of carpal tunnel syndrome in cases where electromyography/nerve conduction studies are inconclusive. This systematic review indicates that US-guided HD techniques are more effective and safer than traditional conservative management. Different types of compounds, such as corticosteroids, NS, LA, PRP, and D5W, have clinical effects, but their mechanisms of action vary. Currently, there is no compound that leads to better outcomes in terms of functional, ultrasonographic, and electrophysiological measures. Therefore, further research is needed to enhance our understanding of US-guided HD procedures for CTS. Large, detailed, randomized, controlled trials are needed to determine the relative safety and effectiveness of this intervention in clinical practice. Future studies should also address other questions related to US-guided HD in CTS, such as the optimal dosages of different injectates and direct comparisons of the effects and efficacy of various injectates.

## Figures and Tables

**Table 1:** General characteristics of included studies, CTS, carpal tunnel syndrome; US, ultrasound; HD, hydrodissection; CSA, cross-sectional area; MN, median nerve; MNI, median nerve injury; VAS, visual analogue scale; NRS; numerical rating scale; BCTQ, Boston carpal tunnel questionnaire; FSS, functional status scale; SSS, symptom severity scale; NCS, nerve conduction studies; SNAP, sensitive nervous action potential; CMAP, compound muscle action potential; NSCV, nerve sensory conduction velocity; DML, distal motor latency; PRP, platelet-rich plasma; D5W, 5% dextrose water; NS, normal saline; HA, hyaluronic acid; PROMs, patient-rated outcome measurements.

Authors	Studied Population	Injectates	Outcomes Evaluated	Follow-Up	Results	Side Effects and Safety Issues
Hsu et al. (2020) [37]	A total of 126 patients with CTS	US-guided lidocaine HD before corticosteroid injection	BCTQ scores; CSA at the inlet of the wrist	2nd week and 6-months	Increase in MN-CSA inlet immediately after HD (>2 mm^2^); no significant difference was observed in BCTQ decreases 2 weeks after the injection between patients with and without MNI	A total of 9 patients with > than 48 h pain and sensitive symptoms after HD (recovered in the 2-week follow-up). A total of 3 patients with vascular injury
Forough et al. (2021) [39]	A total of 40 patients with mild-to-moderate CTS	Corticosteroid or ozone (O2-O3) injection under ultrasound guidance.	VAS and BCTS scores as well as CSA and NCS	After injection and 6th and 12th weeks	Improvement in VAS and BCTS at weeks 6 and 12 after the injections. SNAPs and CMAP latencies and CSA showed significant improvement only among subjects in the corticosteroid group at 6 and 12 weeks	No side-effects or complications
Mezian et al. (2021) [38]	A total of 46 patients were randomly assigned into two groups: group A (perineural injection, 23-group B (peritendinous injection, 23)	A total of 1 mL of trimecaine hydrochloride and 1 mL (40 mg/mL) methylprednisolone acetate	VAS, BCTQ questionnaire, grip strength, 2-point discrimination	12th week	VAS and BCTQ-SSS reached a statistical difference at 2 weeks, while the effect was maintained at 12 weeks. There were no significant differences between follow-up improvements in both groups in the remaining measured parameters (BCTS-FSS, 2-point discrimination, and grip strength)	None of the patients reported adverse events or side-effect
Wang et al. (2021) [47]	A total of 64 patients with CTS	Intra-carpal corticosteroid injection HD group or perineural corticosteroid injection without HD	Primary outcome: BCTQ-SSS score; secondary outcomes: BCTQ-FSS; DML and SNCV.	6th and 12th weeks	Improvement in the SSS and FSS of BCTQ and median nerve DML and SNCV. However, group-by-time interactions were not significant in any outcome measurements.	No serious adverse events, two patients reported minor post-injection pain on the first day after the intervention, which resolved spontaneously
Santoso et al. (2022) [35]	A total of 30 patients with CTS divided into tw groups	Group 1 (*n* = 15) -1 mL triamcinolone acetonide 10 mg/mL + 1 mL lidocaine 2%; group 2 (*n* = 15)-5 mL dextrose 5%	VAS-NRS, BCTQ-FSS, BCTQ-SSS	4 weeks	Significant difference in NRS and BCTQ-FSS and -SSS values at 4 weeks after injection in both groups	-
Li et al. (2020) [41]	A total of 185 patients with a diagnosis of CTS	A total of 10 mL D5W was used for injections in all patients.	PROMs of the injection procedure were categorized into excellent outcome, slight improvement, or no change	15 months	A total of 63 patients were graded as severe. A total of 164 patients reported an effective outcome. Of these, 116 reported an excellent outcome, and 48 reported a good outcome.	No complications in the patients treated with perineural injection
Lin et al. (2020) [36]	A total of 63 patients aged 20–80 years and with idiopathic CTS	Three different volumes of D5W: (1 mL-group; 2 mL-group; 4 mL-group)	Primary outcome: VAS; secondary outcome: BCTQ, QuickDASH, NCS or CSA	24 weeks	Ultrasound-guided HD with 4 mL of D5W provided better efficacy than 1 mL and 2 mL groups based on symptom relief and functional improvement for CTS at the 1st, 4th, and 12th week post injection; no significant difference between the three groups at the 24th-week post-injection follow-up.	No severe adverse effect. No significant difference in the minor symptoms and neuropathic symptoms was observed
He et al. (2022) [40]	A total of 49 patients and 62 wrists with CTS divided into two groups	Combination group—D5W after corticosteroid injection; steroid group (control group)	VAS and BCTQ	Baseline and 4th, 8th, and 12th weeks	Both groups showed greater improvement in VAS, BCTQ-SSS, and BCTQ-FSS at 4-, 8-, and 12-weeks follow-up. Compared with the steroid group, the combination group exhibited a significant reduction in VAS, BCTQ-SSS, and BCTQ-FSS at 8- and 12-week follow-up.	No side effects or complications
Chao et al. (2022) [42]	A total of 36 patients treated for persistent or recurrent CTS after primary surgery	A total of 10 mL of 5% dextrose was used for HD	Primary outcome: % of symptom relief of the affected hand post injection compared to pre injection using VAS and PROMs	33 months	CTS symptoms were categorized as persistent or recurrent in 23/36 and 13/36, respectively. A total of 22/36 patients reported an effective outcome and 14/36 reported a poor outcome. A total of 13%, 39.2%, and 47.8% of patients in the persistent group reported excellent, good, and either minimal or poor outcomes.	No patients reported worsening due to treatment
Wu et al. (2022) [21]	A total of 61 patients with CTS: 15 (NS), 16 (D5W), 15 (PRP), and 15 (HA)	NS, D5W, PRP, and HA	BCTQ-SSS and -FSS, VAS, CSA	6 months	Single doses of PRP, D5W, and HA were more efficient than NS; single injections of PRP and D5W seemed more effective than that of HA within 6 months post injection; for reducing CSA, PRP and HA seemed more effective than D5W; HA was the most effective at the 1st-month post injection and PRP was the most effective at the 6th-month	All patients reported minimal to no pain during and immediately after the procedures, and no adverse event was reported in any patient. None of the recruited patients complained of motor deficit after HD of the MN
Chen et al. (2021) [43]	A total of 26 patients diagnosed with bilateral moderate-to-severe CTS (a total of 52 wrists).	PRP or control groups	Primary outcome: BCTQ scores; secondary outcomes: CSA, NCS	1, 3, 6, and 12 months	PRP group exhibited significant improvements in BCTQ severity scores at all time points, BCTQ functional scores at the 6th month, and cross-sectional area at the 12th month post injection.	-
Chen et al. (2021) [45]	A total of 47 participants with mild-to-moderate CTS.	A total of 5 mL of NS in the two groups (efficacy of short-axis HD to long-axis HD for patients with mild-to-moderate CTS)	Primary outcome: BCTQ-SSS and -FSS; secondary outcome: CSA, NCS	2nd week, and 1, 3, and 6 months	Both groups showed improved BCTQ-SSS, FSS, and CSA at all follow-up assessments; SNCV improved at all follow-up assessments compared to baseline values in both groups; DML was statistically significant at the 6-month follow-up; improvement in 1-month BCTQ-SSS and FSS (short-axis > long-axis group)	No patients showed complications or adverse effects throughout the study
Huang et al. (2023) [34]	A total of 24 patients with CTS	Group 1 (*n* = 12): 10 mL NS; -group 2 (*n* = 12): 5 mL NS	Primary outcome: BCTQ-SSS and -FSS; secondary outcomes: CSA, NCS	4, 12, and 24 weeks	From 0 to 24 weeks, the HD-10 group outperformed the HD-5 group in terms of improvement in SSS and FSS scores; no significant between-group difference was observed in either electrophysiological or CSA measures	No adverse effects were reported or complications observed over the course of the study.
Su et al. (2021) [44]	A total of 40 patients diagnosed with mild or moderate CTS	HA (ultrasound-guided perineural injection of 2.5 mL of HA) or control groups (NS injection) with nerve HD.	Primary outcome: BCTQ scores; secondary outcomes: VAS-NRS, NCS and CSA.	2nd week and 1, 3, and 6 months	Compared with the control group, the HA group did not show significantly superior outcomes, except in terms of BCTQ and NRS at the second week post injection	No adverse events were observed or reported in any patients
Kamel et al. (2020) [46]	A total of 60 patients with chronic CTS	Group 1 (*n* = 30) (HD with hyalase + 10 mL saline solution injection); -group 2 (*n* = 30) (HD with 10 mL NS only)	VAS score, BCTQ-FSS, BCTQ-SSS	Before injection, 1 week, 1 month, 3 months, 6 months	Significantly lower post-injection values of VAS in group 1 versus in group 2, lower BCTQ-FSS scores in group 1 versus in group 2 during all the intervals. Nerve conduction study parameters showed a significantly higher velocity and lower latency in group 1 than in the group 2 by the 3- and 6-month follow-up	-

**Table 2:** Modified version of the Jadad quality scale.

Authors	Was the Treatment Randomly Allocated?	Was the Randomization Procedure Described and Appropriate?	Was There a Description of Withdrawals and Dropouts?	Was There a Clear Description of the Inclusion/Exclusion Criteria?	Were the Methods of Statistical Analysis Described?	Jadad Score (0–5)
Hsu et al., (2020) [37]	No	No	Yes	Yes	Yes	3
Lin et al., (2020) [36]	Yes	Yes	No	Yes	Yes	4
Chen et al., (2021) [45]	Yes	Yes	No	Yes	Yes	4
Wang et al. (2021) [47]	Yes	Yes	No	Yes	Yes	4
He et al. (2022) [40]	Yes	No	No	Yes	Yes	3
Su et al. (2021) [44]	Yes	Yes	Yes	Yes	Yes	5
Chen et al. (2021) [43]	Yes	Yes	No	Yes	Yes	4
Forough et al. (2021) [39]	Yes	Yes	No	Yes	Yes	4
Chao et al. (2022) [42]	Yes	Yes	No	Yes	Yes	4
Li et al. (2020) [41]	Yes	Yes	No	Yes	Yes	4
Mezian et al. (2021) [38]	Yes	Yes	Yes	Yes	Yes	5
Kamel et al. (2020) [46]	Yes	Yes	No	Yes	Yes	4
Santoso et al. (2022) [35]	No	No	No	Yes	Yes	2
Huang et al. (2023) [34]	Yes	Yes	No	Yes	Yes	4
Wu et al. (2022) [21]	Yes	No	No	Yes	Yes	3

## Data Availability

Not applicable.
